# Manganese-Based Targeted Nanoparticles for Postoperative Gastric Cancer Monitoring *via* Magnetic Resonance Imaging

**DOI:** 10.3389/fonc.2020.601538

**Published:** 2020-10-19

**Authors:** Ke Li, Peng Li, Yang Wang, Shuang Han

**Affiliations:** ^1^ Shaanxi Key Laboratory of Brain Disorders, Institute of Basic and Translational Medicine, Xi’an Medical University, Xi’an, China; ^2^ Department of Medical Technology, Xi’an Medical University, Xi’an, China; ^3^ Department of Basic Medical Science, Xi’an Medical University, Xi’an, China; ^4^ Department of Gastroenterology, HongHui Hospital, Xi’an, China

**Keywords:** gastric cancer, contrast agent, magnetic resonance imaging, Mn_3_O_4_, nanoparticles

## Abstract

Postoperative recurrence is a common and severe problem in the treatment of gastric cancer; consequently, a prolonged course of chemotherapy treatment is inevitable. Monitoring by imaging could provide an accurate evaluation of the therapeutic effects, which would be beneficial to guide a treatment strategy adjustment over time. However, current imaging technologies remain insufficient for the continuous postoperative monitoring of gastric cancer. In this case, molecular imaging offers an efficient strategy. Targetable contrast agents are an essential part of molecular imaging, which could greatly enhance the accuracy and quality of monitoring. Herein, we synthesized a Mn-based contrast agent for magnetic resonance imaging (MRI) of gastric cancer monitoring. Initially, small-sized Mn_3_O_4_ nanoparticles (NPs) were synthesized. Then, a functionalized polyethylene glycol (PEG) lipid was attached to the surface of the Mn_3_O_4_ NPs, to improve biocompatibility. The targetable MRI contrast agent (Mn_3_O_4_@PEG-RGD NPs) was further prepared by the conjugation of the arginine-glycine-aspartic acid (RGD) peptides. The completed Mn_3_O_4_@PEG-RGD NPs had the small size of 7.3 ± 2.7 nm and exhibited superior colloidal stability in different solution environments. In addition, Mn_3_O_4_@PEG-RGD NPs exhibited reliable biotolerance and low toxicity both *in vitro* and *in vivo*. Imaging experiments amply demonstrated that Mn_3_O_4_@PEG-RGD NPs could efficiently accumulate in gastric cancer tissues and cells *via* RGD mediation, and immediately significantly increased the MRI effects. Through this study, we can conclude that Mn_3_O_4_@PEG-RGD NPs have the potential to be a novel MRI contrast agent for the postoperative monitoring of gastric cancer.

## Introduction

Gastric cancer currently represents one of the highest incidences of malignant gastrointestinal tumors worldwide. Moreover, the mortality of this disease has increased year over year, with more than approximately 980000 new cases and 730,000 mortalities occurring annually (1–3). Complete prevention of gastric cancer is difficult to achieve. A multitude of factors are related to the disease, such as genetics, dietary habits, environmental elements and bacterial infection (4, 5). The majority of patients with gastric cancer do not exhibit obvious symptoms until the cancer is advanced and cannot be treated effectively. In the clinic, gastrectomy, chemotherapy and radiotherapy are still the main treatments of gastric cancer (6). However, it is important to point out that early diagnosis provides the best opportunity for the effective treatment of gastric cancer because it would provide certain information, such as the location and stage, for initial surgery and radiation treatment. Currently, the methods for the accurate diagnosis of gastric cancer primarily include imaging, detection of biomarkers and tissue biopsy (7, 8). However, postoperative recurrence is very common in mid- and late- stage gastric cancer. This means that a prolonged intense course of chemotherapeutic treatment is necessary. Hence, an accurate assessment of tumor progress is the most significant element to determine the appropriate chemotherapeutic schedule (9, 10). However, some impenetrable limitations and defects in monitoring remain and seriously influence the prognosis (11).

Currently, in the clinical diagnosis of gastric cancer, many imaging technologies, such as endoscopic ultrasonography, computerized tomography (CT), single-photon emission computed tomography (SPECT), positron emission tomography (PET), and MRI, have been demonstrated to be very useful (12, 13). Imaging can reveal the location and border of tumor tissues, which will be used to guide surgery and radiotherapy. However, ionizing radiation, invasive injury, and an expensive cost are inescapable problems during monitoring. Moreover, these defects are more severe in the postoperative monitoring of patients with gastric cancer. Compared with other imaging methods, MRI has certain advantages for the postoperative monitoring of gastric cancer. MRI itself is especially applicable to detection in soft tissues, is a main method in clinical inspection and is characterized by its non-invasiveness, high spatial resolution and radiationless nature (14). This technology could be used to locate and distinguish various tissues for the diagnosis of gastric cancer, but it also has limitations (15). For example, normal MRI lacks the ability to effectively distinguish between food and tumors in the stomach (16). However, molecular imaging provides a novel means to visually access to a tumor at the ultramicro level. This technique has brought significant value gastric cancer monitoring because of its ability to accurately map out tumor tissues in the whole body at the molecular level (17–19). Moreover, molecular imaging not only allows finding the accurate location of the tumors but also possesses the ability to monitor the biological processes of tumor proliferation, metastasis, and response to therapy (20–22). Molecular MRI combines MRI with molecular imaging and has emerged as a novel tool to monitor cancer (23, 24). Functional contrast agents can efficiently enhance the sensitivity of MRI, thereby hopefully solving the present problems (25). Based on the mechanism, the existing MRI contrast agents have broadly been divided into two categories: T1 and T2 contrast agents. T1 contrast agents mainly utilize Gd and Mn. T2 agents are superparamagnetic Fe_3_O_4_ nanoparticles (NPs) (26). Gd-based contrast agents are widely used in clinical MRI. However, there have been some reports of brain deposition and renal fibrosis after the clinical application of Gd-based contrast agent (27, 28). These defects limit the utility of T1 contrast agent for the postoperative monitoring of gastric cancer. Additionally, superparamagnetic Fe_3_O_4_ NPs are being evaluated in clinical tests. However, magnetic susceptibility artifacts and dark signals impede the clinical promotion of T2 contrast agents (29, 30). Mn is a necessary trace element that has a high relaxation spin and bright signal; thus, Mn-based contrast agents have attracted considerable attention in recent decades (31). However, Mn is difficult to use in MRI directly. A suitable Mn preparation might help overcome this limitation.

**Figure 1:**
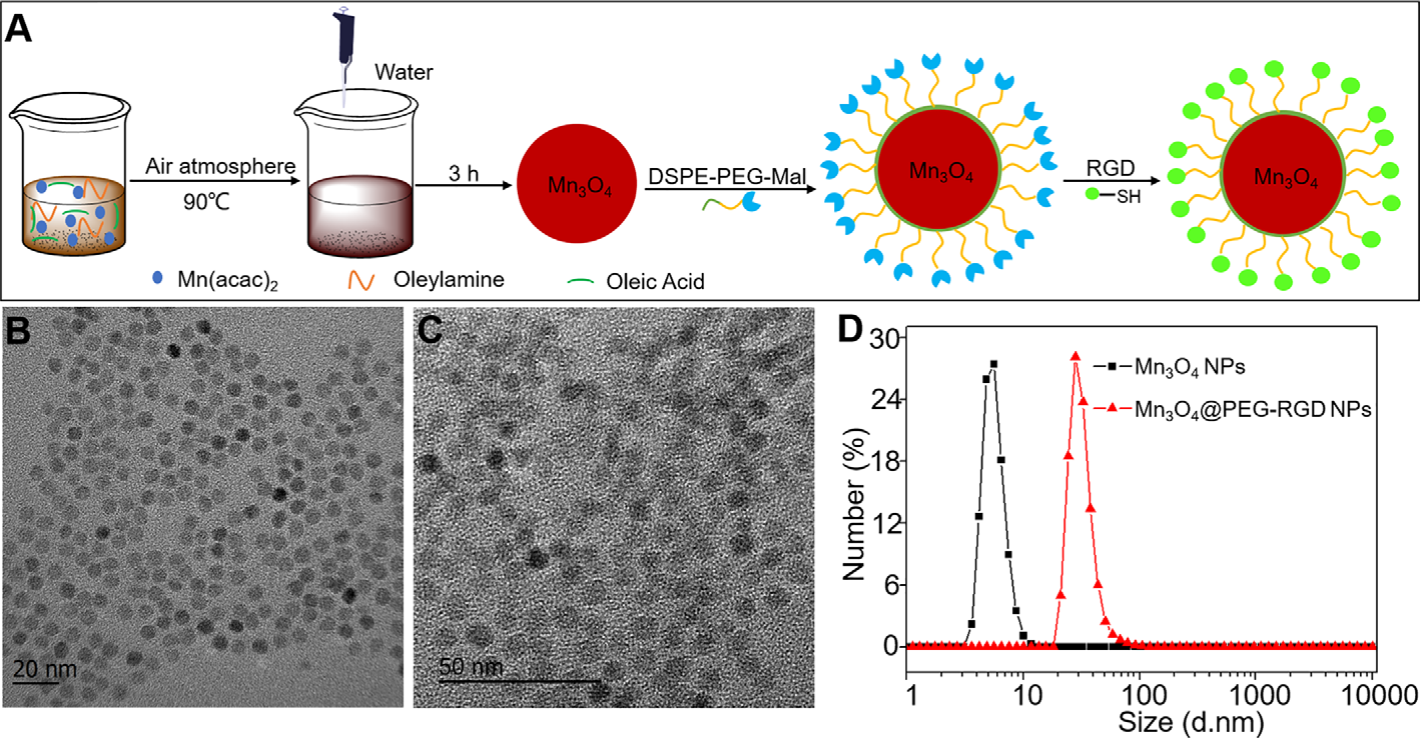
Synthesis and characteristics of Mn_3_O_4_@PEG-RGD NPs. The preparation scheme of Mn_3_O_4_@PEG-RGD NPs **(A)**, TEM observations of Mn_3_O_4_ NPs **(B)**, and Mn_3_O_4_@PEG-RGD NPs **(C)**; Distributions of hydrodynamic size of Mn_3_O_4_ NPs and Mn_3_O_4_@PEG-RGD NPs **(D)**.

NPs could provide an effective opportunity to address the above-mentioned problem. The NPs could be loaded with a functional agent as an enhanced beacon for imaging. A novel NPs-based contrast agent could improve the MRI effects for the diagnosis of gastric cancer, which allows earlier and more accurate detection of tumor tissues and improves the prognosis of the disease. Mn_3_O_4_ can be used to synthesize NPs with favorable monodispersity. The synthesis conditions are mild and have high yields (32). Many Mn_3_O_4_-based contrast agents have been reported for tumor imaging, including MRI, a combination of fluorescence and MR and an MR/PET combination (33–35). Currently, there are researchers who are exploiting more effective Mn_3_O_4_ NPs contrast agents. In this study, we synthesized small-scale Mn_3_O_4_ NPs to monitor gastric cancer *in vivo*. However, Mn_3_O_4_ NPs cannot disperse in the aqueous phase and lack a positive tumor target. In order to overcome these defects, we applied functionalized polyethylene glycol (PEG) lipids to modify the Mn_3_O_4_ NPs. PEG was modified on the surface of Mn_3_O_4_, which allowed the NPs to stably disperse in water. The functional groups in PEG also allow for the further conjugation of targeting molecules. The RGD peptide was employed as the targetable component in this case. The RGD peptide possesses ligand-receptor interaction with αvβ_3_ integrin, which is a transmembrane protein that mediates interactions between the inside and outside of living cells (36). Integrin αvβ_3_ is expressed at low level in normal cells but is usually over-expressed in a wide variety of cancer cells, such as gastric cancer, breast cancer and hepatic cancer (37–40). Thus, RGD-based targeted nanocarriers have been developed for tumor treatment (41). It is crucial for Mn_3_O_4_ NPs to actively target gastric tumor for MRI. The synthetic process is exhibited in **Figure 1A**, and the final MRI contrast agent was named Mn_3_O_4_@PEG-RGD NPs. The NPs possess low toxicity, effective biocompatibility and T1-weighted imaging, which could be utilized for the postoperative monitoring of gastric cancer.

## Materials and Methods

### Materials

Manganese (II) acetate (98%), oleic acid, oleylamine, and fluorescein isothiocyanate isomer (FITC) were purchased from Aladdin Inc. (Shanghai, China). DSPE-PEG-NH_2_, DSPE-PEG-Mal, and SCM-PEG-Mal and supplied by Creative PEGworks (NC, USA). Core molecule was PEG5000. Traut’s reagent and RGD antibody were bought from Thermo Fisher Inc. (MA, USA). RGD peptide was purchased from Haode Peptide Co., Ltd. (Wuhan, China). Human umbillical vein endothelial cells (HUVECs), human embryonic lung fibroblast cells (IMR-90), human gastric adenocarcinoma cells (SGC-7901), human gastric carcinoma cells (BGC-823), DMEM high glucose medium, and foetal bovine serum (FBS) were purchased from Procell Inc. (Wuhan, China). The CCK-8 kit, DAPI kit, antibiotics and trypsin (0.25%) were ordered from Beyotime Inc. (Shanghai, China). Other chemical reagents were purchased from Sinopharm Corp. (Beijing, China). BALB/c mice and BALB/c-nu/nu mice were purchased from Peking HFK Biotech Corp. (Beijing, China).

### Synthesis of Mn_3_O_4_ NPs

The synthesis of Mn_3_O_4_ NPs was described in Yu’s report (32). First, 1 mmol of manganese acetate (0.17 g), 640 μl of oleic acid and 3.28 ml of oleylamine were added to 15 ml of xylene, and the mixture was heated to 90°C with stirring. The mixture reacted for 10 min. Then, 1 ml of ultrapure water was added to the mixture, and the reaction continued for another 2.5 h with vigorous stirring. When the reaction was complete, 40 ml of ethyl alcohol was added to the mixture. The powdered Mn_3_O_4_@PEG-Mal NPs were obtained *via* centrifugation.

### Synthesis of Mn_3_O_4_@PEG-RGD NPs

Ten milligrams of Mn_3_O_4_ NPs powder were dispersed into 3 ml of chloroform, and 25 mg of DSPE-PEG5000-Mal was added. The mixture stirred for 4 h after which chloroform was removed *via* rotary evaporation. The mixture was eluted with 10 ml of ultrapure water under sonication for 30 min, and excess DSPE-PEG-Mal was removed *via* centrifugation. Thus, Mn_3_O_4_@PEG-Mal NPs was obtained. In the next step, Traut’s reagent and a solution of the peptide RGD (molar ratio of 1:25) were co-incubated at pH 8.0 for 2 h. Then, the Mn_3_O_4_@PEG-Mal NPs were added to the solution dropwise and the mixture was further incubated with stirring under the same conditions for 1 h. Excess peptide and Traut’s reagents were removed by 3 cycles of centrifugation. Thus, complete Mn_3_O_4_@PEG-RGD NPs were obtained for further experiments.

FITC-labeled Mn_3_O_4_@PEG-RGD NPs were also synthesized. In the first step, Mn_3_O_4_@PEG-NH_2_ NPs was prepared by the same process of Mn_3_O_4_@PEG-Mal NPs. Then Mn_3_O_4_@PEG-NH_2_ NPs were conjugated with FITC at pH 8.5 for 3 h. The molar ratio of FITC to Mn_3_O_4_@PEG-NH_2_ NPs was 1:10. The NPs were further conjugated with SCM-PEG-Mal under the same conditions as those of DSPE-PEG-NH_2_ conjugation. Finally, FITC-Mn_3_O_4_@PEG-Mal NPs was obtained. The RGD peptide was then conjugated onto the NPs *via* the same method which mentioned previously.

### Characterization of Mn_3_O_4_@PEG-RGD NPs

The hydrodynamic sizes of the Mn_3_O_4_ NPs and Mn_3_O_4_@PEG-RGD NPs were measured with a Malvern Zetasizer Nano ZS (Malvern, UK). Transmission electron microscopy (TEM) was used to investigate the morphologies and sizes of the NPs. The X-ray diffraction (XRD) pattern of the Mn_3_O_4_ NPs was investigated with Cu Kα radiation (λ = 0.15405) with a Bruker D8 diffractometer (MA, USA). The T1-relaxivities and T1-weighted images of Mn_3_O_4_ aqueous solution were observed and measured by a 0.5 T mouse MRI scanner (Niumag Corp., Shanghai, China). The conventional spin-echo acquisition sequence was as follows: TE = 18.2 ms, TR = 350 ms, Slice Thickness = 4 mm, and Slice Gap = 0.8 mm. The stability of the NPs was evaluated by measuring their hydrodynamic sizes under different conditions. In order to detect whether the NPs could be used in further *in vitro* or *in vivo* investigations, the dispersion solutions included PBS, complete medium, and FBS.

### Cell and Animal Models

Two normal human cell lines (HUVECs and IMR-90 cells) and two human gastric cancer cell lines (SGC-7901 and BGC-823 cells) were utilized in the study. All cell lines were incubated in DMEM high glucose medium with 10% of FBS, 1% of antibiotics at 37°C under 5% CO_2_. Logarithmic phase cells were seeded into dishes or plates for further utilization in *in vitro* experiments.

BALB/c-nu/nu mice were used to establish xenograft animal model. Four-week-old male mice were fed under SPF conditions 5 days for acclimation. If the physiological status of the mice was normal, they could be used for model. First, 100 μl of a SGC-7901 cell suspension containing 1 ×10^6^ cells was subcutaneously injected into the right crotch of each mouse. When the tumor grew to a sufficient size, the animal could be used for *in vivo* experiments. All animal experiments were supervised by the Laboratory Animal Administration Committee of Xi’an Medical University. Animal experimental protocols followed by the Guidelines for the Use and Care of Experimental Animals at Xi’an Medical University.

### Cytotoxicity Tests

The *in vitro* cytotoxicity of Mn_3_O_4_@PEG-RGD NPs was evaluated by CCK-8 assay. Four human cell lines (HUVECs and IMR-90, SGC-7901 and BGC-823 cells) were used for the evaluation of the *in vitro* cytotoxicity. Mn_3_O_4_@PEG NPs and RGD peptide were used as controls. During the logarithmic growth phase, cells were seeded into 96-well plate at a density of 8 × 10^4^ cells/well. Subsequently, samples at different concentrations were added. After 72 h, when the cells in the untreated wells grew to 90% confluence, the medium was replaced with fresh colorless medium containing 10% CCK-8 agent. The plate was further incubated for 2 h, and the absorbance at 450 nm of each well was measured with a microplate reader (Infinite^®^ 200 Pro, Tecan, Switzerland). The cell viability was calculated.

### Cell Internalization and Affinity Assay

FITC-labeled Mn_3_O_4_@PEG-RGD NPs were prepared for evaluation by internalization and affinity assays. SGC-7901 cells were incubated in confocal dishes for 24 h. Then, the NPs were added to the dishes. Subsequently, the cells were fixed with a 4% paraformaldehyde solution for 0.5 h, 2 h and 4 h, and then the cell nuclei were stained with a DAPI kit. The dishes were observed *via* a confocal microscope (TCS SP5 II, Leica, Germany). The affinity assay was utilized to evaluate targeted delivery *in vitro*. The RGD peptide was the competitive agent of NP endocytosis. Cells were again incubated in confocal dishes for 24 h. Half of the dishes were supplemented with the RGD peptide as a blocking agent. After 1 h, the NPs were added. After incubation, the dishes were fixed with a 4% paraformaldehyde solution, and the cell nuclei were stained with DAPI. The dishes were observed by confocal microscopy. All fluorescent signals were quantified by ImageJ software.

### Hemolysis Assay

The primary route of administration of the Mn_3_O_4_@PEG-RGD NPs is intravenous injection. Therefore, the influence of the NPs on erythrocytes should be evaluated. Whole blood was drawn from the mice, and heparin was immediately added. The red blood cells were collected by centrifugation. The cells were then resuspended in PBS at 2% concentration. The red blood cell suspension was infused into a 6-well plate, and then Mn_3_O_4_@PEG-RGD NPs, Mn_3_O_4_@PEG NPs and RGD were added. Trition X-100 (1%, v/v) and saline were the positive and negative controls, respectively. The plates were incubated at 37°C for 2 h. Subsequently, the cell suspensions were centrifuged, and then the supernatant was collected for determination of the absorbance at 394 nm.

### *In Vivo* Acute Toxicity

Thirty of BALB/c mice (15 females and 15 males, with an average weight of 20 g) were fed under SPF conditions for 5 days to acclimatize themselves, and then, they were randomly divided into 3 groups. The three groups were intravenously injected with Mn_3_O_4_@PEG-RGD NPs, Mn_3_O_4_@PEG NPs or RGD peptide. The doses of both the Mn_3_O_4_@PEG-RGD NPs and Mn_3_O_4_@PEG NPs groups were 100 mg/kg, and the dose of RGD peptide was 2 mg/kg. The survival rate was recorded within 14 d. Subsequently, the remaining mice were euthanized, and their organs were collected for pathological evaluation.

### *In Vivo* MRI Investigation

Three xenograft mice were used for the MRI experiment. Two mice were intravenously injected with either Mn_3_O_4_@PEG-RGD NPs or Mn_3_O_4_@PEG NPs. The third mouse was administrated 2 mg of the RGD peptide intratumorally for blocking and then intravenously injected with Mn_3_O_4_@PEG-RGD NPs. The doses of both the Mn_3_O_4_@PEG-RGD NPs and Mn_3_O_4_@PEG NPs groups were 30 mg/kg. T1-MRI was performed with a 0.5 T mouse MRI scanner (Niumag Corp., Shanghai, China). The sequential time points of imaging were 0, 0.5, 1, 2, 4, and 8 h. The MRI system parameters were as follows: TE = 18.2 ms, TR = 350 ms, Slice Thickness = 4 mm, Flip Angle = 90°, FOV = 100, NEX = 2, Matrix: 256 × 256, Axial images.

### Histology Assay

A xenograft mouse was intravenously injected with Mn_3_O_4_@PEG-RGD NPs at a dose of 30 mg/kg. After 4 h, the mouse was euthanized, the liver, kidney, spleen and tumor were collected. All tissues were frozen and immunofluorescent staining for histological assays. The RGD antibody and FITC-labeled second antibody were used to mark the Mn_3_O_4_@PEG-RGD NPs. The slides were observed with an inverted fluorescence microscope (DP72, Olympus, Tokyo, Japan).

### Statistical Analysis

GraphPad Prism 5.0 software was employed to calculate the data. The data of independently repeated experiments are presented as the mean values ± standard deviation (SD). Statistical differences between the groups are indicated p < 0.05.

## Results

### Preparation and Characterization of the Mn_3_O_4_@PEG-RGD NPs

The process of Mn_3_O_4_@PEG-RGD NPs preparation is exhibited in **Figure 1A**. The synthesis included three steps: synthesis of the Mn_3_O_4_ NPs, modification with functional PEG and then conjugation with the RGD peptide. **Figures 1B, C** are TEM images of the Mn_3_O_4_ NPs and Mn_3_O_4_@PEG-RGD NPs. Both kinds of NPs were spherical and showed good monodispersity. The diameters of the Mn_3_O_4_ NPs and Mn_3_O_4_@PEG-RGD NPs were 5.4 ± 1.4 nm and 7.3 ± 2.7 nm, respectively. The increase in size preliminarily indicated that the surface modification was successful. Further, the hydrodynamic diameters of Mn_3_O_4_ NPs and Mn_3_O_4_@PEG-RGD NPs were measured with a Malvern instrument. The average sizes of the NPs are shown in **Figure 1D**. The size of the Mn_3_O_4_ NPs was 5.9 ± 1.9 nm, showing no obvious differences between the TEM observation. Nevertheless, in the measurement of Mn_3_O_4_@PEG-RGD NPs, the hydrodynamic size was 28.5 ± 7.4 nm, which was more than 4 times that of the TEM observation. The primary reason for this result is that the hydrated PEG layer of the Mn_3_O_4_@PEG-RGD NPs evaporated during sample preparation and TEM observation. The Mn_3_O_4_ NPs were dispersed in cyclohexane since the NPs could not be dispersed in aqueous solution. By comparison, PEG modified NPs could be effectively dispersed in water. The results also indicated that PEG successfully crosslinked onto the surface of the Mn_3_O_4_ NPs.

**Figure 2:**
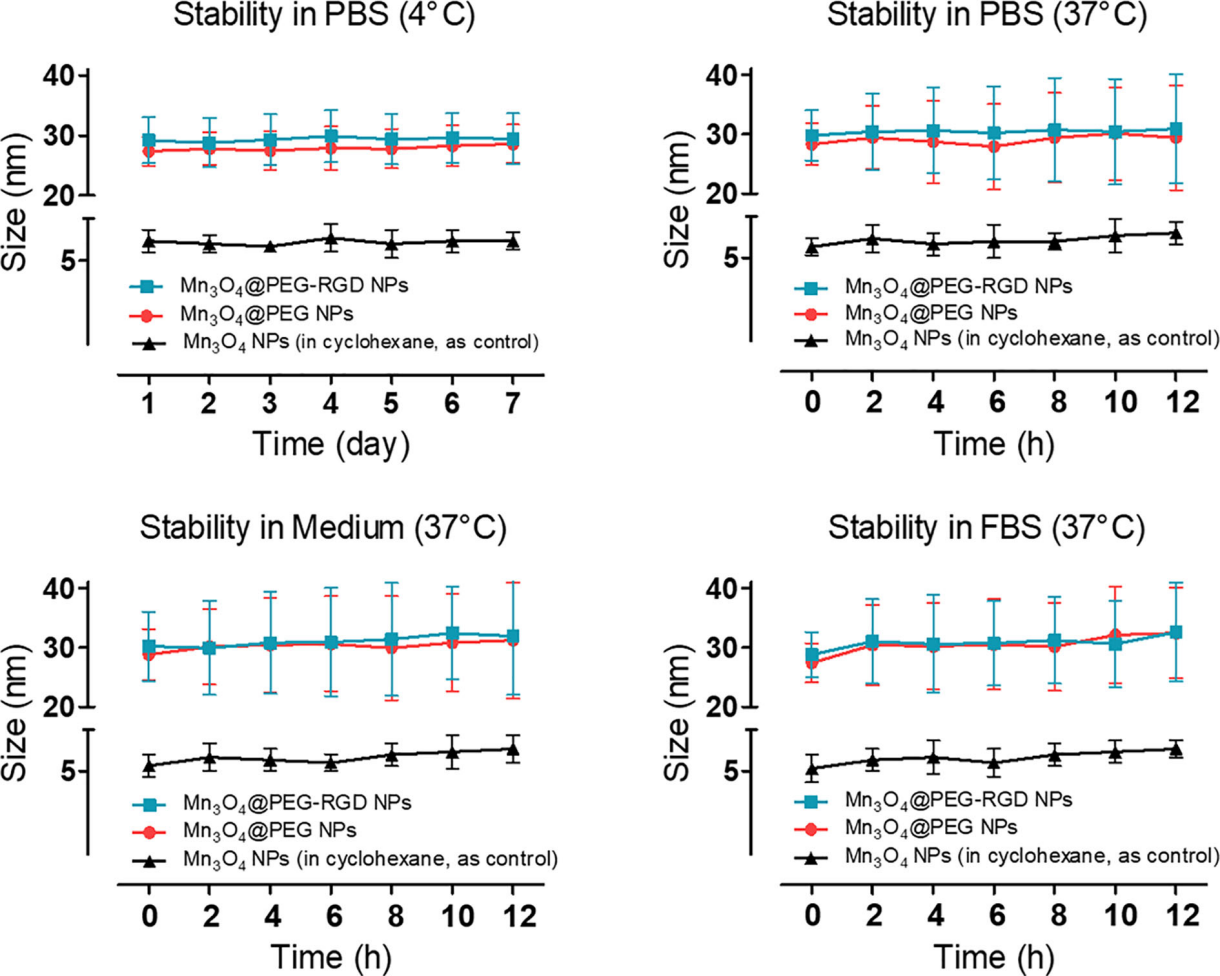
Colloidal stability of Mn_3_O_4_@PEG-RGD NPs. The NPs were dispersed in PBS, complete medium and FBS, respectively. Mn_3_O_4_ NPs and Mn_3_O_4_@PEG NPs were employed as control. Mn_3_O_4_ NPs was dispersed in cyclohexane.

The XRD pattern indicates that the Mn_3_O_4_ NPs were well-crystallized. The Joint Committee on Powder Diffraction Standard (JCPDS) card number 24-0734 was used to contrast the diffraction peaks. The results are shown in **Figure S1**. The Mn_3_O_4_ NPs peaks coincide with the standard. The stability of the NPs was evaluated by changing the hydrodynamic size under different conditions. Mn_3_O_4_ NPs were dispersed in cyclohexane as the control. The results are shown in **Figure 2**. Both the Mn_3_O_4_@PEG NPs and Mn_3_O_4_@PEG-RGD NPs could be stably dispersed in PBS. Compared with the control, the size distributions of Mn_3_O_4_@PEG NPs and Mn_3_O_4_@PEG-RGD NPs at 37°C were significantly broader than those at low temperature. The possible reason for this result is that an increase in temperature promotes PEG to form a thicker hydrated rete. Similarly, the Mn_3_O_4_@PEG NPs and Mn_3_O_4_@PEG-RGD NPs, which were dispersed in medium and FBS, also showed broad size distribution curves. Beside temperature factor, proteins in solution appear to have an impact on hydrated retia. Overall, Mn_3_O_4_@PEG NPs and Mn_3_O_4_@PEG-RGD NPs could be effectively dispersed in various conditions, indicating good colloidal stability of the NPs.

**Figure 3:**
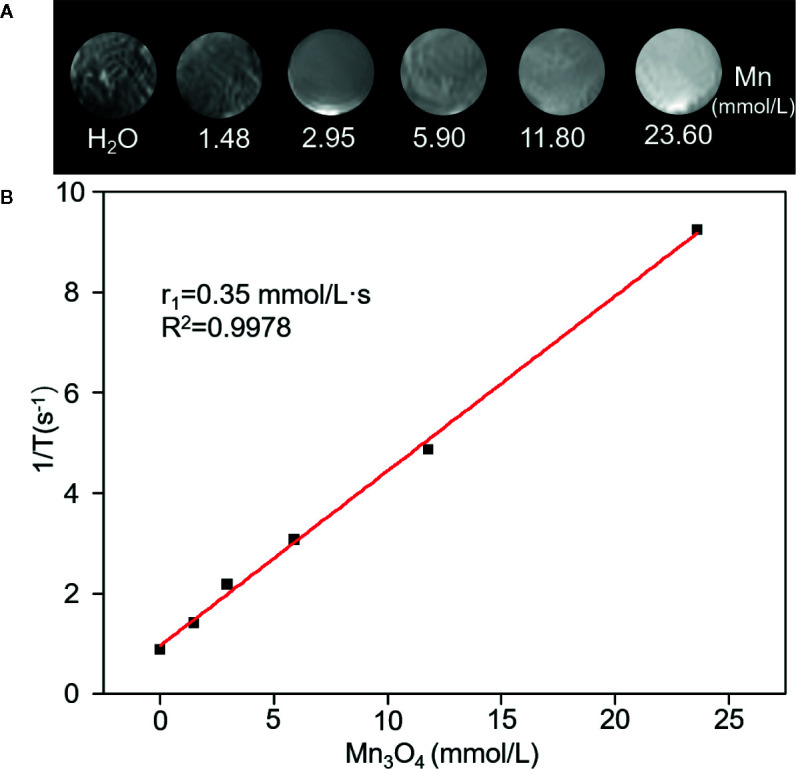
Relaxation performances of Mn_3_O_4_@PEG-RGD NPs. T1-weighted MRI of the NPs in aqueous phase **(A)**. Calculation of Relaxation rate (*r*_1_) of Mn_3_O_4_@PEG-RGD NPs **(B)**.

A 0.5 T MRI scanner was utilized to evaluate magnetic resonance contrast performance of Mn_3_O_4_@PEG-RGD NPs in aqueous solution. As shown in **Figure 3A**, Mn_3_O_4_@PEG-RGD NPs exhibited a significant reinforcement effect on T1-MRI. The concentration-response relationship is remarkable. The relaxation rate (*r*_1_) of the Mn_3_O_4_@PEG-RGD NPs was calculated by measuring the proton relaxation time. The results are shown in **Figure 3B**. The data also correspond to the concentration of the NPs. The *r*_1_ value was determined to be 0.35 mmol/L·s.

### *In Vitro* Cytotoxicity and Internalization Assay

**Figure 4:**
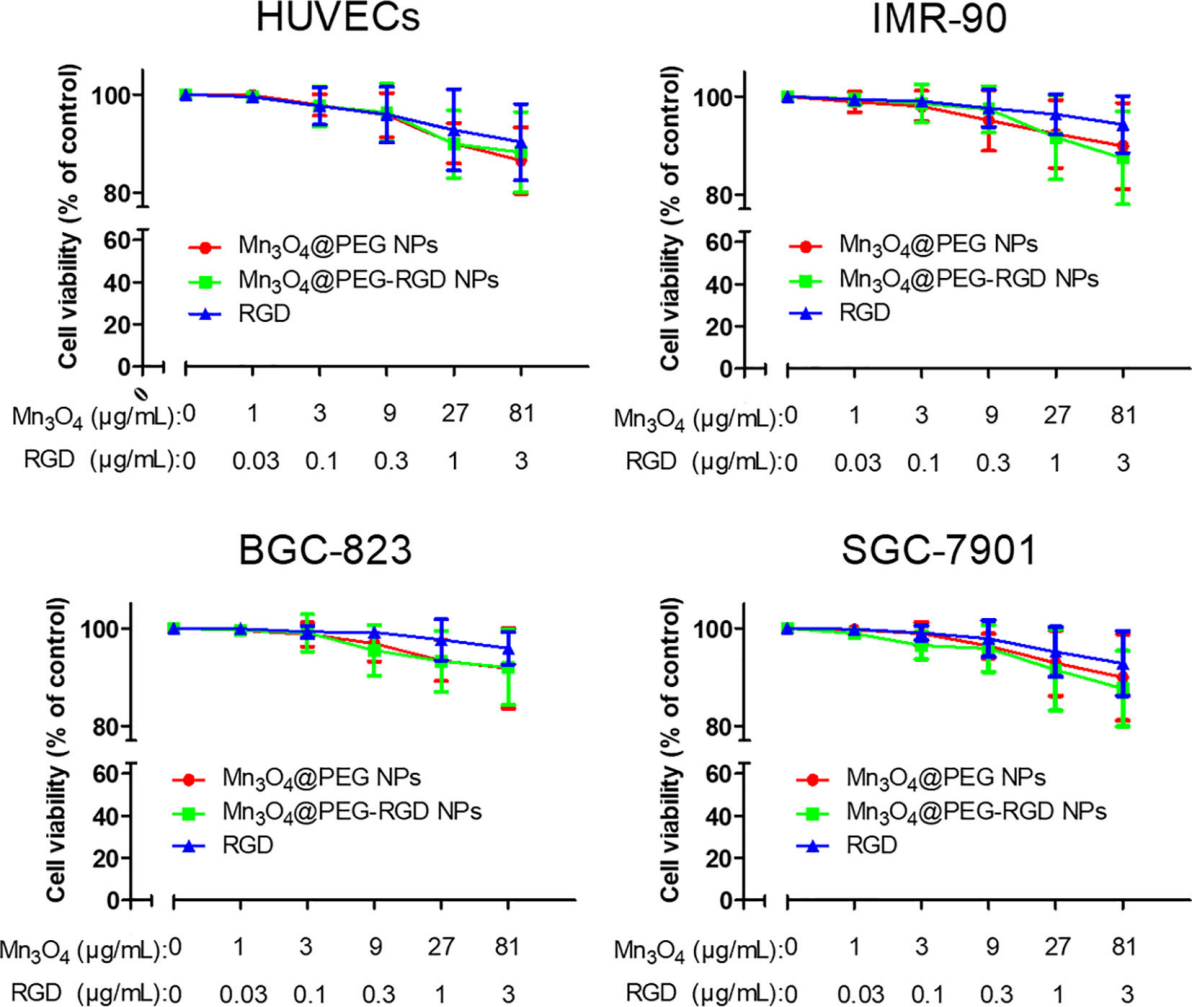
*In vitro* cytotoxicity of Mn_3_O_4_@PEG-RGD NPs. Cell viabilities of HUVECs, IMR-90, BGC-823, and SGC-7901, which were incubated with different concentrations of the NPs. Mn_3_O_4_@PEG NPs and RGD peptide were used as control. The concentration of NPs was set by quantity of Mn_3_O_4_.

The cytotoxicity of Mn_3_O_4_@PEG-RGD NPs was evaluated by CCK-8 assay, which used two normal human cell lines (HUVECs and IMR-90 cells) and two of human gastric cancer cell lines (SGC-7901 and BGC-823 cells). Mn_3_O_4_@PEG NPs and the RGD peptide were used as the control. Mn_3_O_4_ NPs were not applicable in this experiment due to their water-insoluble nature. As shown in **Figure 4**, all of those treatments exhibited low cytotoxicity in 4 cell lines. More than 90% of the cells survived at the highest concentration of Mn_3_O_4_. The cell viabilities did not exhibit significant difference between treatment with the Mn_3_O_4_@PEG-RGD NPs and Mn_3_O_4_@PEG NPs. The results preliminarily indicate that Mn_3_O_4_@PEG-RGD NPs have good biocompatibility *in vitro*.

**Figure 5:**
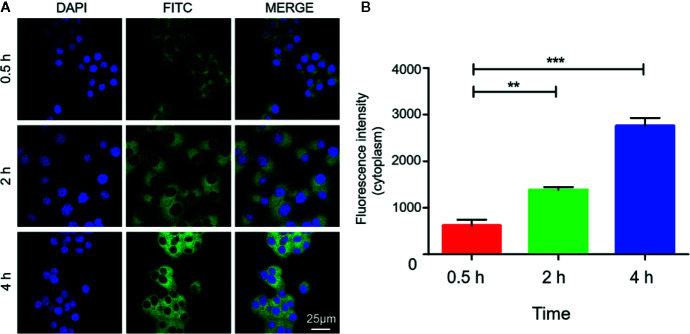
Internalization of Mn_3_O_4_@PEG-RGD NPs in SGC-7901 cell line. Confocal imaging of cell co-incubation in different time points **(A)**. Quantitation of fluorescent intensity in cell **(B)**. Error bars represent the SD of the mean. The ** indicated p < 0.01, *** indicated p < 0.001.

**Figure 6:**
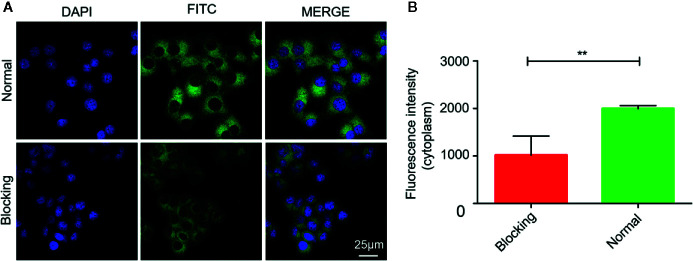
RGD blocking test of Mn_3_O_4_@PEG-RGD NPs. Confocal imaging of cell in different treatments **(A)**. Quantitation of fluorescent intensity in cell **(B)**. Error bars represent the SD of the mean. The ** indicated p < 0.01.

The internalization process of Mn_3_O_4_@PEG-RGD NPs is shown in **Figure 5**. The green and blue fluorescence originates from FITC-labeled Mn_3_O_4_@PEG-RGD NPs and DAPI-labeled cell nuclei respectively. An enhancement in cellular green fluorescence reflects the internalization process. After the initial 0.5 h, the green fluorescent signal appeared in the cytoplasm. Then the fluorescence intensity gradually increased in cells. After 2 h, the cell image appeared completely. At 4 h, the fluorescent signal strength in the cell accumulated to a higher level. The RGD peptide provided the NPs cancer cell targeting function, which was achieved by the interaction with the integrin receptor. In order to verify the effect of targeted delivery, we used the RGD peptide as a competitive inhibitor to block Mn_3_O_4_@PEG-RGD NPs endocytosis. The results are shown in **Figure 6**. The green fluorescent intensity in blocking group was significantly lower than that in the untreated group, which means the RGD peptide could effectively block the internalization of Mn_3_O_4_@PEG-RGD NPs. This result indicates that the transmembrane transport of Mn_3_O_4_@PEG-RGD NPs is mediated by RGD-integrin receptor interactions and preliminarily suggests that Mn_3_O_4_@PEG-RGD NPs could achieve tumor-targeted delivery.

### *In Vivo* Toxicity Evaluation

**Figure 7:**
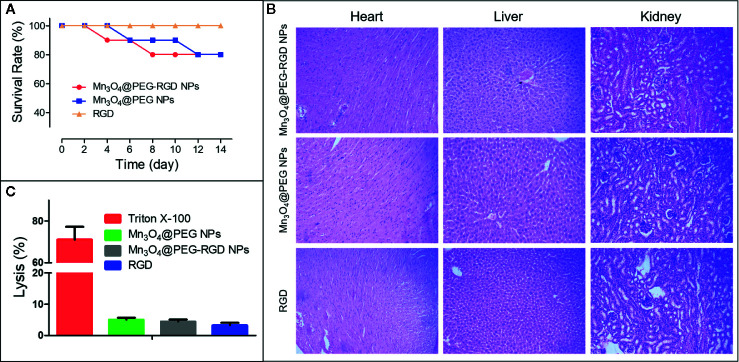
*In vivo* toxicity of Mn_3_O_4_@PEG-RGD NPs. Survival rate of mice in different treatments **(A)**. Pathological sections of heart, liver and kidney **(B)**. Results of hemolytic assay **(C)**.

The route of administration of Mn_3_O_4_@PEG-RGD NPs is intravenous injection. Therefore, the erythrocyte impact of the NPs should initially be evaluated. The hemolysis results are shown in **Figure 7C**. Triton X-100 caused severe red blood cell plasmorrhexis, and the lysis rate was over 70%. By comparison, the samples in the Mn_3_O_4_@PEG-RGD NPs, Mn_3_O_4_@PEG NPs, and RGD treatment groups exhibited low lysis rates, all of which were less than 5%. The results indicate that Mn_3_O_4_@PEG-RGD NPs have little impact on erythrocytes. The safety of intravenous injection is guaranteed.

Subsequently, an acute toxicity test was further employed to evaluate the *in vivo* toxicity of the Mn_3_O_4_@PEG-RGD NPs. As **Figure 7A** shows, no mice died in the RGD peptide treatment group, and most of the mice survived in the Mn_3_O_4_@PEG-RGD NPs and Mn_3_O_4_@PEG NPs groups, which did not exhibit prominent toxic effects at a high dose. The pathological sections further demonstrated the results. The NPs treatments did not obviously injure the main organs. Histological evaluation of the heart, liver and kidney did not show pathological injury (**Figure 7B**). These results amply demonstrated that Mn_3_O_4_@PEG-RGD NPs had excellent biocompatibility.

### *In Vivo* MRI Evaluation

**Figure 8:**
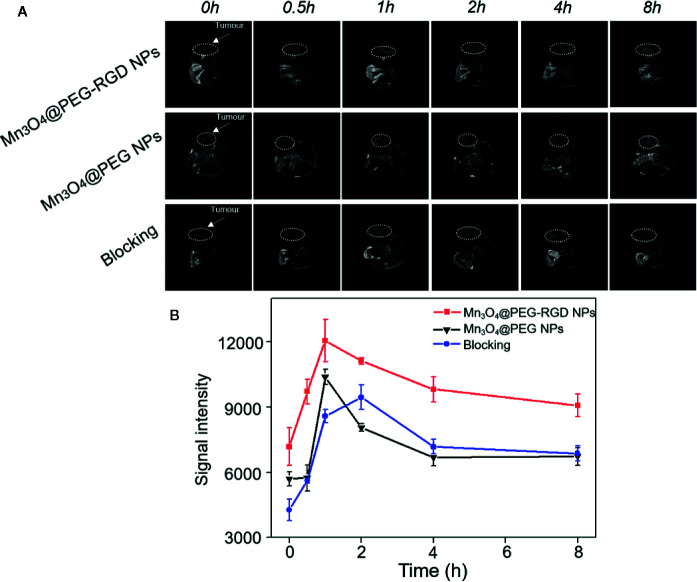
*In vivo* T1-weighted MRI of Mn_3_O_4_@PEG-RGD NPs at different time points. White circles points location of tumor **(A)**. Quantitation of MR intensity in tumor at different time points **(B)**. Error bars represent the SD of the mean.

The BALB/c nu/nu mouse xenograft model was utilized to evaluate the T1-weight effect of Mn_3_O_4_@PEG-RGD NPs in MRI. First, the NPs were intravenously injected into each mouse. The images were then successively captured in sequential time points. The MRI images are shown in **Figure 8A**, and the MR signal intensity was calculated and is exhibited in **Figure 8B**. In the Mn_3_O_4_@PEG-RGD NPs group, the signal was observed immediately. The reason for this result is that the scanning time of MRI needs approximately 25 min; and at this time, the NPs have accumulated in the tumor tissues. After 1 h, the signal in the Mn_3_O_4_@PEG-RGD NPs group reached its maximum, and then fell gradually. Mn_3_O_4_@PEG NPs did not show positive targeted performance, and the accumulation quantity in the tumor was obviously lower than that of the Mn_3_O_4_@PEG-RGD NPs group. Nevertheless, both NPs treatment groups exhibited similar trends. The curve in the RGD-blocking treatment group showed difference that the peak appeared in 2 h, and the intensity was stronger than that in the Mn_3_O_4_@PEG NPs group in subsequent 6 h. This trend indicates that RGD could initially block the positive tumor target of Mn_3_O_4_@PEG-RGD NPs. When the RGD peptide is metabolized, the targeted delivery of Mn_3_O_4_@PEG-RGD NPs is regained.

**Figure 9:**
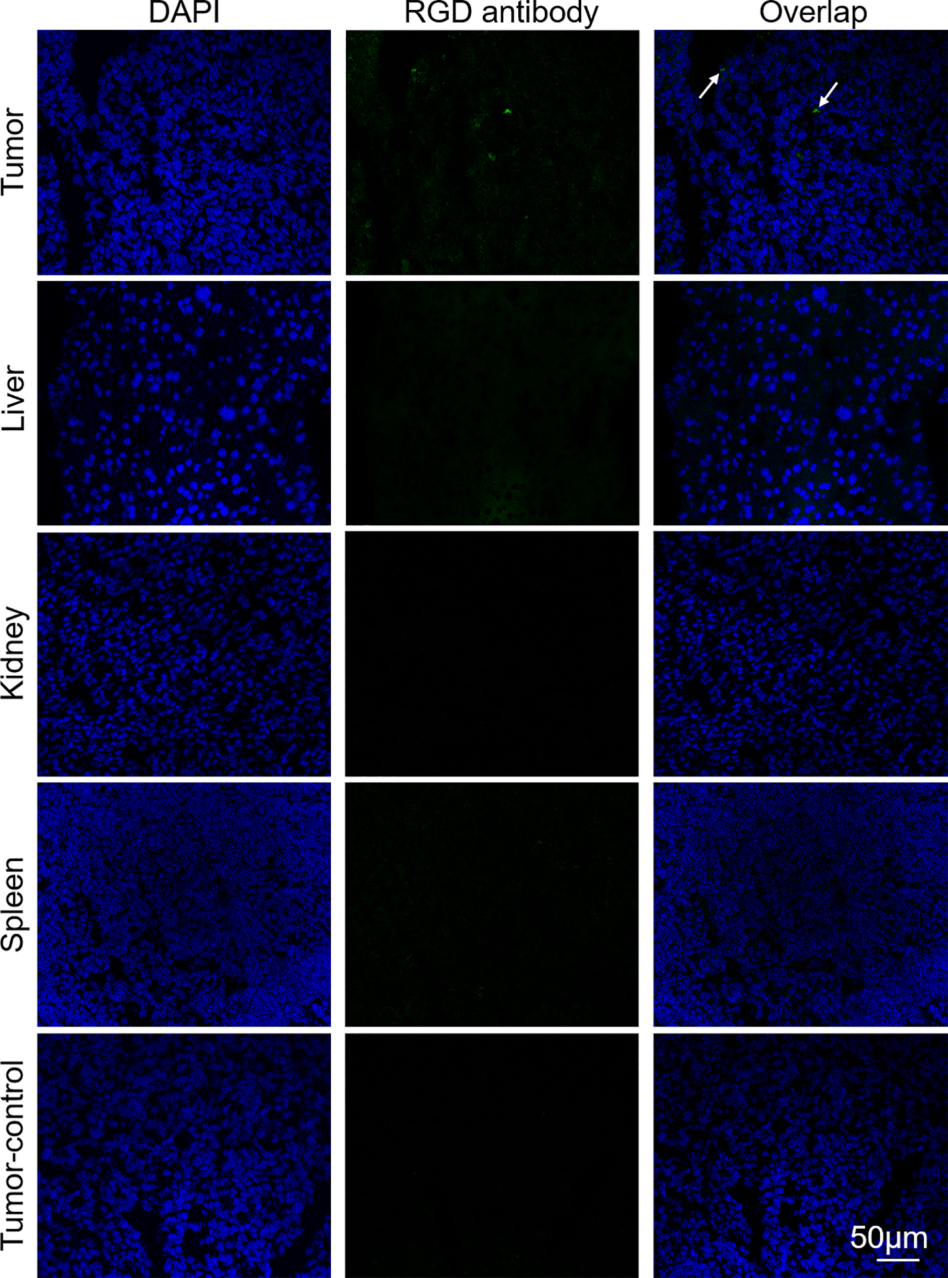
Immunofluorescent assay of Mn_3_O_4_@PEG-RGD NPs in various tissues *via* RGD antibody. Green fluorescent signal derives Mn_3_O_4_@PEG-RGD NPs (Pointed by white arrow). Blue fluorescent signal is from DAPI, which indicates nuclei. Scale bar: 50 μm.

Immunofluorescence analysis was further employed to verify the targeted delivery of the Mn_3_O_4_@PEG-RGD NPs. As shown in **Figure 9**, green fluorescent spots were observed in tumor tissues from the Mn_3_O_4_@PEG-RGD NPs treated mouse obviously. However, the fluorescent spot was almost invisible in the normal organs after Mn_3_O_4_@PEG-RGD NPs treatment and in the tumor after Mn_3_O_4_@PEG NPs treatment. These results further demonstrate that Mn_3_O_4_@PEG-RGD NPs have an excellent tumor targeting function.

## Discussion

Worldwide, gastric cancer has become one of the most common malignant gastrointestinal tumors and displays high mortality (1). In gastric cancer, radical surgery can lead to a positive curative effect. However, postoperative recurrence is an unavoidable problem in the majority of patients. If recurrence occurs, chemotherapy is the primary treatment, and the therapy must be long-term. How effectively patients continue their therapy can also depend on the evaluation of the therapeutic effect, especially the imaging-guided monitoring. To prolong survival in patients with gastric cancer, researchers employed various imaging technologies for diagnosis. Although, gastroscopy, CT, PET, and SPECT exhibit accuracy in the early diagnosis of gastric cancer, radioactivity and invasive injury severely limit their applications in continuous monitoring. By comparison, MIR, a non-invasive and non-radiological imaging method, is an important iconography for gastric cancer diagnosis (14). Contrast agents can be used to further improve clarity in MRI scans of the cancer tissues. However, there are currently still several problems with the administration of contrast agents in clinical MRI. In order to increase the efficiency of the MRI in continuous postoperative monitoring, we prepared Mn_3_O_4_@PEG-RGD NPs, which could be used for *in vivo* gastric cancer MRI observation. The core of the contrast agent is a Mn_3_O_4_ NPs. Synthesis of the NPs was based on the thermal decomposition reaction of water and manganese acetate in the presence of oleylamine. During the synthesis, water promoted the nucleation of nanocrystals, and oleylamine was used as catalyst (32). As the results show, Mn_3_O_4_ NPs were spherical and appeared to be monodispersed in an excellent manner. The average size of the NPs was approximately 5 nm. This result was also verified by XRD. However, the relatively low biocompatibility of Mn_3_O_4_ NPs was a problem. The NPs dispersed in cyclohexane, meaning that they could not be used in the aqueous phase. In order to compensate for this limitation, we employed PEG to modify the Mn_3_O_4_ NPs (25, 42, 43). Moreover, for further modification, a functionalized PEG lipid was used in the hydrophilization of the Mn_3_O_4_ NPs. The Mn_3_O_4_@PEG NPs also exhibited good monodispersity and aqueous stability. Their size in TEM observation increased by approximately 2 nm, which indicated that PEG successfully covered the surface of Mn_3_O_4_ NPs. In the next step, the RGD peptide was further conjugated to the PEG layer, which provided a tumor-targeting function. The hydrodynamic size of the Mn_3_O_4_@PEG-RGD NPs also increased nearly quintuple. This phenomenon was due to the expansion of the PEG hydration layer in the DLS measurements (44, 45). Colloidal stability is a critical performance indicator of the NPs (46). Dispersed in PBS, medium and FBS, the sizes of the Mn_3_O_4_@PEG-RGD NPs or Mn_3_O_4_@PEG NPs were sequentially measured. The Mn_3_O_4_@PEG-RGD NPs did not show flocculation and precipitation throughout the experiment. More notably, the data indicated that the hydrodynamic size of the NPs was very stable in different solutions and under various temperature conditions. These results amply demonstrated that the NPs exhibited excellent stability under physiological conditions. Importantly, the manufacturing process and cost of the Mn_3_O_4_@PEG-RGD NPs are efficient enough for mass production and application. These NPs will be more beneficial for the continuous postoperative monitoring in patients with gastric cancer.

Biotolerance, which ensures safe *in vivo* application, is a necessary feature for MRI agents. In order to evaluate whether the Mn_3_O_4_@PEG-RGD NPs could be used as an MRI contrast agent, a series of experiments were conducted. Initially, the *in vitro* cytotoxicity of the NPs was evaluated in four cell lines, including HUVECs and IMR-90, SGC-7901 and BGC-823 cells. HUVECs are human umbilical vein endothelial cells and used to evaluated the impact of the NPs on the blood vessel endothelium. Originating from human embryonic lung fibroblast tissues, IMR-90 cells were used to verify the cytotoxicity of Mn_3_O_4_@PEG-RGD NPs in normal cell. Both SGC-7901 and BGC-823 cells were employed to investigate their influence on gastric cancer cells. The results of the CCK-8 assay indicated that the Mn_3_O_4_@PEG-RGD NPs did not show obvious cytotoxicity in any of the four cell lines. The cell viabilities were over 90%, even at maximum concentration of the Mn_3_O_4_@PEG-RGD NPs. A similar result appeared after treatment with Mn_3_O_4_@PEG NPs. Therefore, the low cytotoxicity of the Mn_3_O_4_@PEG-RGD NPs was verified. Subsequently, we evaluated *in vivo* toxicity of Mn_3_O_4_@PEG-RGD NPs. Initially, a hemolysis assay was applied to investigate whether the Mn_3_O_4_@PEG-RGD NPs could impact red blood cells. The lysis rate after treatment with Mn_3_O_4_@PEG-RGD NPs was less than 5%. Then, we further evaluated the *in vivo* acute toxicity in animals. Mn_3_O_4_@PEG-RGD NPs, Mn_3_O_4_@PEG NPs and RGD peptide were injected into BALB/c mice, respectively. The intravenous injection dose was 100 mg/kg, which is well above the dose for practical imaging applications. After two weeks of observation and recording, 80% of the mice in Mn_3_O_4_@PEG-RGD NPs treatment groups were survived. By comparison, the survival ratios in the Mn_3_O_4_@PEG NPs and RGD peptide groups were also impressive. In addition, pathological analysis further verified that there were no obvious injuries to the major organs. Based on these results, the biotolerance of the Mn_3_O_4_@PEG-RGD NPs was amply demonstrated. And it indicated that the NPs could be safely utilized in long-term for MRI *in vivo*. The excellent performance suggests that Mn_3_O_4_@PEG-RGD NPs could be used for the continuous postoperative monitoring in gastric cancer patients, without cumulative toxicity.

MR imaging of Mn_3_O_4_@PEG-RGD NPs is the essential endpoint of this study. A series of *in vitro* and *in vivo* imaging experiments were employed to evaluate whether Mn_3_O_4_@PEG-RGD NPs could be used as an MRI contrast agent for the postoperative monitoring of gastric cancer. In the evaluation of magnetic resonance contrast performance, Mn_3_O_4_@PEG-RGD NPs showed a remarkable concentration-response relationship. As the concentration increased, the MRI signal was enhanced. The *r*_1_ value was 0.35 mmol/L·s. The data are much lower than that of Gd-based MR contrast agents. The probable reason for the low *r*_1_ value might be due to the valences of Mn_3_O_4_. Mn_3_O_4_ contains two trivalent Mn atoms and one bivalent Mn. The paramagnetic strength of the ions depends on the number of unpaired electrons in the 3d orbital. Thus, more unpaired electrons will have stronger paramagnetic strength (31). The unpaired electron number of the trivalent Mn is less than that of divalent Mn; therefore, the *r*_1_ value of Mn_3_O_4_ is relatively low. Besides, PEG modification also probably contributes to this result. PEG provides hydrophilia to the Mn_3_O_4_ NPs. At the same time, it forms a thick hydrophobic layer, which hinders the chemical exchange between magnetic ions and protons (47, 48). However, Mn_3_O_4_@PEG-RGD NPs still exhibited obvious T1-weighted effects both *in vitro* and *in vivo*. It can fully meet the demands of the monitoring of gastric cancer. The gastric tumor target of Mn_3_O_4_@PEG-RGD NPs was mediated by RGD-integrin receptor interaction (36). The integrin receptor is widely expressed on the cytomembrane of various tumor cells, such as in gastric cancer, hepatic cancer, pulmonary cancer and colon cancer (37, 39, 40). In this study, fluorescently labeled NPs were used for the observation of internalization (49, 50). Under normal conditions, FITC-labeled Mn_3_O_4_@PEG-RGD NPs gradually accumulated in the cytoplasm of SGC-7901 cells. However, competitive suppression with the RGD peptide could block the endocytosis of Mn_3_O_4_@PEG-RGD NPs. The results preliminarily demonstrated that the NPs possess gastric tumor targeted delivery. Subsequently, the *in vivo* MRI evaluation of Mn_3_O_4_@PEG-RGD NPs was performed in a gastric cancer xenograft mouse model. In Mn_3_O_4_@PEG-RGD NPs-treated mouse, the MR signal was rapidly observed in tumor tissues after intravenous injection. After 1 h, the intensity of MR signal reached its peak, and then gradually decreased. The signal in Mn_3_O_4_@PEG NPs was significantly lower than that in the targeted NPs. Remarkably, intratumoral injection of the RGD peptide could effectively blocking accumulation of Mn_3_O_4_@PEG-RGD NPs. Thus, Mn_3_O_4_@PEG-RGD NPs have an excellent *in vivo* gastric tumor-targeting ability and can effectively perform *in vivo* MR imaging. Moreover, immunofluorescence analysis verified that Mn_3_O_4_@PEG-RGD NPs could accumulate in gastric tumor in the mouse model, meanwhile, barely exhibited accumulation in normal organs and tissues. The results indicate that Mn_3_O_4_@PEG-RGD NPs can rapidly penetrate into the gastric tumor, and then be gradually metabolized. In principle, the metabolites are safe and non-toxic. The RGD peptide will be hydrolyzed in the same manner as other amino acids. PEG is widely used in medicine and food. Mn is a necessary element in the body that can effectively be metabolized and excreted out of the body. Throughout the whole monitoring process, the Mn_3_O_4_@PEG-RGD NPs did not cause obvious side effects, which could certify their long-term reliability. All of these results indicated that Mn_3_O_4_@PEG-RGD NPs may have great potential for the MRI postoperative monitoring of gastric cancer.

## Conclusion

In the present study, we demonstrated that Mn_3_O_4_@PEG-RGD NPs could be a reliable MRI contrast agent for monitoring gastric cancer *in vivo*, due to its possession of obvious advantages, such as simple preparation, low cost, effective T1-weighted, rapid metabolism, and low toxic. All of these attributes are suitable for the long-term monitoring of gastric cancer. Initially, Mn_3_O_4_ NPs were synthesized followed by modification with PEG and conjugation of the RGD peptide to prepare the targeted T1-MRI contrast agent. Within these NPs, the Mn_3_O_4_ NPs are the core for MRI. An appropriate size and shape make the NPs usable in MRI and suitable for further modification and functionalization. PEG modification makes these NPs to disperse in aqueous solution, thus resolving the problem of biocompatibility. The RGD peptide provides the NPs with a gastric tumor-targeting function, which allows the NPs to rapidly accumulate in gastric tumor tissues *in vivo*. Moreover, a series of evaluations verified that Mn_3_O_4_@PEG-RGD NPs have excellent biosecurity and colloidal stability. In conclusion, Mn_3_O_4_@PEG-RGD NPs were amply demonstrated to be a potential nano contrast agent for postoperative monitoring of gastric cancer *via* MRI.

## Data Availability Statement

The raw data supporting the conclusions of this article will be made available by the authors, without undue reservation.

## Ethics Statement

The animal study was reviewed and approved by Laboratory Animal Administration Committee of Xi’an Medical University.

## Author Contributions

KL and SH designed the study. KL and PL performed the experiments. KL, PL, YW, and SH analyzed the results and data. KL and PL prepared the manuscript. YW and SH modified the manuscript. All authors contributed to the article and approved the submitted version.

## Funding

This study was supported, in part, by the National Natural Science Foundation of China (81801863), the Natural Science Basic Research Program of Shaanxi (2019JQ-485), Innovation Capability Support Program of the Shaanxi Province (2020KJXX-050).

## Conflict of Interest

The authors declare that the research was conducted in the absence of any commercial or financial relationships that could be construed as a potential conflict of interest.

## Supplementary Material

Supplementary Figure 1 X-ray diffraction pattern of Mn_3_O_4_ NPs.
