# Supplementary figures and images for: Manganese-Based Targeted Nanoparticles for Postoperative Gastric Cancer Monitoring via Magnetic Resonance Imaging
Source: Front Oncol. 2020 Oct 19;10:601538. doi: 10.3389/fonc.2020.601538 (PMC7604458; doi:10.3389/fonc.2020.601538)

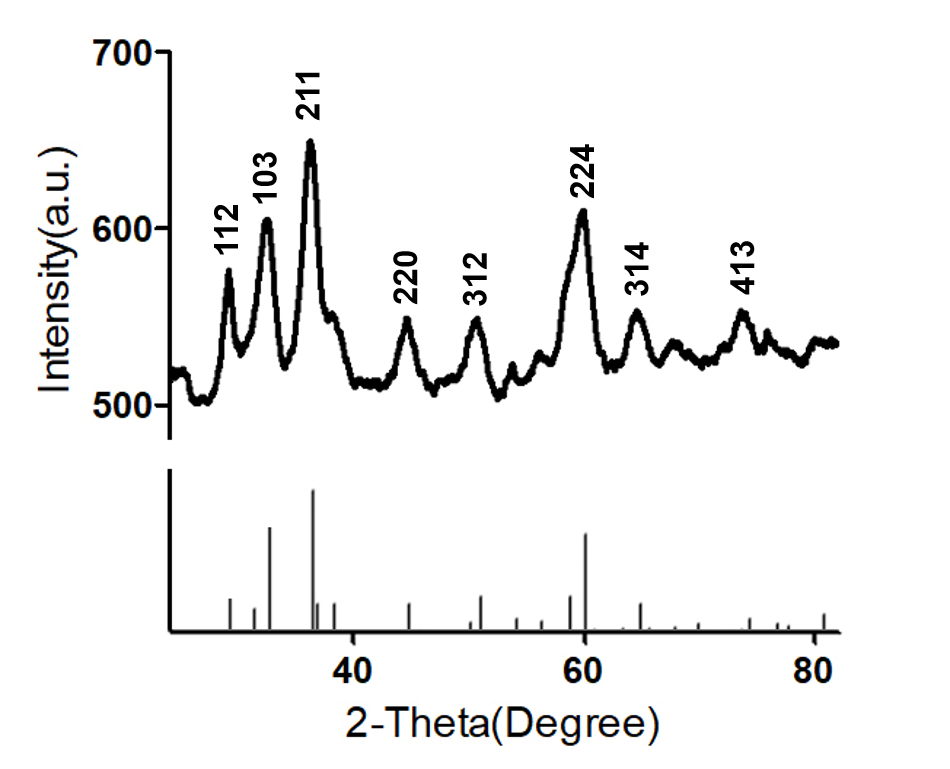

Supplement: Supplementary Figure 1 — X-ray diffraction pattern of Mn3O4 NPs. [file Image_1.tif]
